# Supplementary material for: Mechanisms governing the pioneering and redistribution capabilities of the non-classical pioneer PU.1
Source: Nat Commun. 2020 Jan 21;11:402. doi: 10.1038/s41467-019-13960-2 (PMC6972792; doi:10.1038/s41467-019-13960-2)
Supplement: Supplementary file 7 — Source data [file 41467_2019_13960_MOESM7_ESM.zip › Source_Data/FigureS2/FigureS2B_MotifScanOutput/KmeansCluster11/homerResults/motif4.similar.html]

motif4

## Information for motif4

T
G
A
C
C
T
G
A
A
T
G
C
A
G
C
T
C
G
A
T
T
G
A
C
G
A
T
C
A
G
C
T
A
C
G
T
A
T
G
C
A
G
T
C
  
Reverse Opposite:  

C
T
A
G
T
A
C
G
G
T
C
A
T
C
G
A
C
T
A
G
A
C
T
G
G
C
T
A
T
C
G
A
T
A
C
G
G
A
C
T
A
C
T
G
  

|  |  |
| --- | --- |
| p-value: | 1e-138 |
| log p-value: | -3.192e+02 |
| Information Content per bp: | 1.691 |
| Number of Target Sequences with motif | 503.0 |
| Percentage of Target Sequences with motif | 15.48% |
| Number of Background Sequences with motif | 1704.1 |
| Percentage of Background Sequences with motif | 3.77% |
| Average Position of motif in Targets | 103.0 +/- 51.5bp |
| Average Position of motif in Background | 101.6 +/- 70.4bp |
| Strand Bias (log2 ratio + to - strand density) | 0.6 |
| Multiplicity (# of sites on avg that occur together) | 1.02 |
| Motif File: | file (matrix) reverse opposite |

### Similar de novo motifs found

|  |  |  |  |  |  |  |  |
| --- | --- | --- | --- | --- | --- | --- | --- |
| Rank | Match Score | Redundant Motif | P-value | log P-value | % of Targets | % of Background | Motif file |
| 1 | 0.923 | C T A G A C T G C G T A C T G A C T A G T C A G T G C A C T G A A T C G C A G T C T A G C A G T | 1e-47 | -109.499822 | 8.62% | 3.01% | motif file (matrix) |
| 2 | 0.787 | C G T A G T A C A T G C G T C A G T A C A G C T A G C T G T A C A G T C C G T A A G T C A G T C A G T C | 1e-24 | -56.157832 | 1.23% | 0.10% | motif file (matrix) |
| 3 | 0.798 | G A T C G T A C A G T C G A T C A G C T A T G C G A T C A G C T C A G T A T G C G A T C G A T C A G T C A G C T | 1e-14 | -32.539582 | 2.28% | 0.75% | motif file (matrix) |
| 4 | 0.809 | T G A C G T C A A T G C A G T C A G C T A T G C A G T C A C G T A C G T G T A C A G T C A G C T C G A T A T G C | 1e-12 | -28.380480 | 1.79% | 0.55% | motif file (matrix) |
